# How do nurses and teachers perform breast self-examination: are they reliable sources of information?

**DOI:** 10.1186/1471-2458-7-96

**Published:** 2007-06-05

**Authors:** Fatma Demirkiran, Nevin Akdolun Balkaya, Sakine Memis, Gulengun Turk, Safiye Ozvurmaz, Pars Tuncyurek

**Affiliations:** 1 Psychiatric Nursing, Adnan Menderes University School of Health,Genclik Cad. No:7, 09100 Aydin / Turkey; 2 Gynecological Nursing, Adnan Menderes University School of Health, Genclik Cad. No:7, 09100 Aydin / Turkey; 3 Medical Nursing, Adnan Menderes University School of Health, Genclik Cad. No:7, 09100 Aydin / Turkey; 4 Fundamentals of Nursing, Adnan Menderes University School of Health, Genclik Cad. No:7, 09100 Aydin / Turkey; 5 Public Health Nursing, Adnan Menderes University School of Health, Genclik Cad. No:7, 09100 Aydin / Turkey; 6 Department of Surgery, Adnan Menderes University Faculty of Medicine, Genclik Cad. No:7, 09100 Aydin / Turkey

## Abstract

**Background:**

Breast cancer is the most common cause of cancer-related deaths among women worldwide. The aim of the present study was to determine and compare knowledge, behavior and attitudes among female nurses and teachers concerning breast self-examination (BSE).

**Methods:**

Two-hundred and eighty nine women working in Aydin, Turkey (125 nurses and 164 teachers) were included in the study. The data were collected using a questionnaire designed to measure the knowledge, attitudes and behavior of the groups. Analysis involved percentiles, χ^2 ^tests, *t *tests and factor analysis.

**Results:**

The knowledge of nurses about BSE was higher than that of teachers (81.5% versus 45.1%; p < 0.001). BSE practice parameters (i.e. age groups, indications, frequency) were similar (p > 0.05), whereas skills in performing self-examination were higher in nurses (p < 0.001). Fear of having breast cancer is the most frequent reason for performing BSE. Among nurses, the reasons for failure to perform BSE were the absence of prominent breast problems (82%) and forgetting (56.4%). The teachers who did not perform BSE said that the reasons were lack of knowledge on how to perform self-examination (68.9%) and absence of problems (54%). Both groups had unacceptable technical errors in the performance of BSE.

**Conclusion:**

We conclude that nurses and teachers should be supported with information enabling them to accomplish their roles in the community. To improve BSE practice, it is crucial to coordinate continuous and planned education.

## Background

Breast cancer is the most common cause of cancer-related deaths among women worldwide [1-3]. It accounts for 31% of cancers among women, and 19% of deaths among women are due to cancer [1]. Epidemiological data show that one in 8 women in the United States of America and one in 10 women in Europe will develop breast cancer at some time during their lives [4,5].

Breast cancer is the most prevalent form of cancer in Turkey as well, accounting for 24.1–26.7% of all female cancers [6,7]. Studies of breast self-examination (BSE) in Turkey have shown that the percentage of women who knew how to perform BSE ranged from a low of 9.9% to a high of 45.9% [8,9]. Kırdök et al. [10] and Öncel et al. [11] suggest that 41.8% of Turkish women working in the health care sector know when to perform BSE and 67.1% know how to perform BSE.

Although breast cancer is one of the most common reasons for death among women, diagnosis at an earlier stage of the disease (i.e. tumors less than 2 cm in diameter) allows women more treatment choices and a greater chance of long-term survival [12,13]. Imaging studies that are recommended for early detection of breast cancer (i.e. screening mammography) cannot be routinely applied in countries with restricted health service resources [14-16].

The early detection and diagnosis rates of breast cancer are considerably lower among Turkish women than among women in Western countries [17]. Mammography is not a routine part of regular medical examination in Turkey; it is used for patients at higher risk of breast cancer [18]. Therefore, many women miss early detection and treatment opportunities owing to lack of information, knowledge and awareness of breast cancer, as well as to cancer screening practices [19]. Most cases are diagnosed in advanced stages, so it is compulsory to raise awareness of breast cancer screening in the community [12,18].

Recent reports suggest that BSE is a reliable screening tool when used as an adjunct to clinical breast examination and imaging studies [12,20,21]. The Guidelines of the American Cancer Society also encourage BSE for early detection of breast cancer in asymptomatic women [13]. Therefore, we consider BSE a reliable self-screening tool for the early diagnosis of breast cancer.

It has been reported that early diagnosis of breast cancer is related to the frequency of BSE [22]. Kern also emphasized the unique value of BSE. It has been suggested that more than three quarters of patients with delayed diagnosis initially report a self-discovered breast mass, although further evaluation does not yield a positive mammogram [23]. In accordance with the common notion that a negative mammogram does not exclude the possibility of malignancy, our national management plan for a suspected breast mass is to obtain immediate core biopsies from the self-discovered mass to avoid delayed diagnosis [6]. Some authors do not recommend routine BSE training since it does not decrease breast cancer mortality and causes unnecessary breast biopsies [24-26]. Despite these findings, BSE remains a valuable screening method that also increases awareness in the community. The Canadian Cancer Society [27] and the American Cancer Society [13] continue to advise all women over 20 years old to perform regular BSE. By these means, women become familiar with the normal appearance and feel of their breasts and are better able to recognize changes and report them to their doctor for further professional evaluation [14,28,29].

Nurses play a unique role in alerting the community to the early detection of breast cancer as they usually have the closest contacts with female patients [12,30,31]. Nurses can use their knowledge of the health services to educate women about breast cancer risk factors and available breast cancer screening services and practices [32]. School teachers may also play an important role in health education, helping young people to develop healthy behavior including BSE. In health education, students should gain an understanding and appreciation of healthy lifestyles that promote lifelong wellbeing [33-36].

The main aim of the present study was to determine and compare the knowledge, behavior and attitudes among female nurses and teachers in regard to breast self-examination. To our knowledge, no national data have so far been published on this subject. The research question addressed in this empirical investigation was: Do nurses and teachers differ in their knowledge and behavior about, and attitudes towards, breast self-examination?

## Methods

This was a cross-sectional analytic study performed in Aydin, a small western city located in the Aegean region of Turkey with a population of 903,677 (2004 census).

The local government authorities and managerial offices approved the study. Participants received BSE instruction after filling in the questionnaire; it took 15 minutes to answer the whole questionnaire. Nurses and teachers received BSE instruction in separate groups.

### Participants

Nurses working in Adnan Menderes University Hospital and Aydin State Hospital (n = 125) were included in the study. Female teachers (n = 164) working in primary and high schools in the State were evaluated as the second group. Informed consent was obtained from all participants.

There were 125 (43.2%) nurses and 164 (56.7%) teachers in the study. The mean ages of the nurses and teachers were 31.8 ± 7.2 and 39.8 ± 6.7 respectively (t = -9.71, p = 0.0001). Work experiences were 10.8 ± 7.25 years for nurses and 17.1 ± 6.90 years for teachers.

Of the nurses, 52.9% had associateships and 32.5% had bachelor degrees; 74.4% were married and 67.2% had children. Of the teachers, 93.8% had bachelor degrees; 83.4% were married and 79.8% had children (Table 3).

**Table 3:** Demographic data of nurses and teachers

**Demographic**	**Nurse**		**Teacher**	
	n^a^	%	n^a^	%
Education				
High school	18	14.6	-	-
Associate degree	65	52.9	10	6.2
Bachelor	40	32.5	152	93.8

Total	123	100.0	162	100.0

Marital status				
Married	93	74.4	136	83.4
Single	32	25.6	27	16.6

Total	125	100.0	163	100.0

Child				
Yes	84	67.2	130	79.8
No	41	32.8	33	20.2

Total	125	100.0	163	100.0

Longest inhabited in a				
Village	8	6.5	3	1.8
Town	6	4.8	8	4.9
City	110	88.7	152	93.3

Total	124	100.0	163	100.0

^a ^n value differs because of unanswered questions

### Data collection and collation

Data were collected by a questionnaire comprising three sections, which was designed in line with the relevant literature [2,14,18,21,37-39].

### First section

#### Socio-demographic characteristics

Demographic data (age, marital status, parental status, education, working period, location of longest period of residency) were obtained in this section.

#### Breast cancer history

Family history of breast cancer was sought.

### Second section

#### BSE knowledge and practice

The questions intended to measure knowledge about BSE, together with the correct answers, are shown in Table 1.

**Table 1:** The questions and corresponding answers about knowledge of BSE

**BSE Knowledge**	**The answers accepted as "true"**
• Who should perform BSE?	• *Both male and female individuals*.
• When should a girl begin BSE?	• *19 years old or older*.
• How often should BSE be performed?	• *Monthly*.
• When should a woman with regular menstruations do BSE?	• *Two days after the cessation of menstruations*.
• When should a woman with irregular menstruations do BSE?	• *A regular day of each month*.
• What will be the position of body while performing BSE?	• *Standing (in front of the mirror) or lying*.
• Which examination technique should be applied during BSE?	• *Inspection and palpation (using any of circular, radial, or vertical methods, with the palmer side of three middle fingers. The contact should be continuous)*.

Answers concerning knowledge and behavior in regard to BSE were evaluated as "true" or "false" according to the criteria defined by Smith et al. and The Turkish National Family Planning Guideline [38,39].

### Third section

#### BSE attitudes

In order to measure attitudes towards BSE, 19 statements were used (Table 2). Fourteen were designed by the investigators in accordance with the needs of this study. Five were taken from a form used by Budden [37]:*(1) I am too busy to do breast self-examinations; (2) The thought of breast cancer scares me; (3) Discovering lumps early would increase my chance of survival if I had breast cancer; (4) Breast self-examination can help me find lumps in my breast; (5) I feel that I will get breast cancer in the future*. These five questions were translated from English into Turkish by two academician nurses. Two clinical psychologists who are fluent in English checked the translations for linguistic and conceptual equivalence. Participants rated the 20 attitude items on a 5-point Likert scale ranging from *strongly disagree *(1) to *strongly agree *(5). In order to determine the factor structure of the 20 attitude items, a Varimax-rotated principal components analysis (Varimax with Kaiser Normalization) was carried out and yielded five factors that explained 63% of the total variance. Factor scores were computed by summing the responses to the items under each factor and dividing by the number of items in the respective factor. Thus, factor scores ranged from 1 to 5. The internal consistency reliability coefficient (Cronbach's *alpha*) for the total scale was 0.73. Factor names, loadings, eigenvalues and internal consistency coefficients are shown in Table 2. As Table 2 shows, the internal consistency coefficients for factors 4 and 5 are low. These low internal consistency coefficients imply that factors 4 and 5 are not reliable for use as independent subscales. Therefore, we eliminated them from further analyses.

**Table 2:** Varimax rotated factor analysis of attitudes towards BSE

**Factor names and items**	**Factor loading**
***1. The importance for the BSE in the early detection of breast cancer (eigenvalue = 4.5; variance = 14.18; α = 0.98)***	
Discovering lumps early would increase my chance of survival if I had breast cancer	0.89
BSE is important for early diagnosis of breast cancer	0.87
Breast self-examination can help me to find lumps in my breast,	0.84
***2. Fear of Breast cancer (eigenvalue = 2.5; variance = 27.27; α = 0.81)***	
The thought of breast cancer scares me	0.85
The possibility of breast cancer frightens me	0.83
If I had breast cancer, I would feel bad	0.80
I am afraid of having breast cancer in future	0.50
***3. Positive attitude to BSE (eigenvalue = 2.0; variance = 40.02; α = 0.81)***	
I look for new information on breast examination	0.73
If I ever have breast cancer, I can struggle with this disease	0.57
I can check the progression of a mass in my breast with BSE	0.55
I think I have to examine my breasts regularly	0.55
I think every woman should regularly (monthly) perform BSE	0.54
I think nurses and teachers should advise their patients and students to perform BSE	0.51
***4. Avoidance of BSE (eigenvalue = 1.3; variance = 50.67; α = 0.68)***	
Monthly breast examination embarrasses me	0.80
I am too busy to do breast self-examination	0.77
Performing BSE is disturbing	0.71
I think it is wrong to touch my body that way	0.49
***Factor 5. Breast cancer risk perception (eigenvalue = 1.2; variance = 57.78; α = 0.23)***	
It is probable that I will have breast cancer in future	0.78
I feel that I will get breast cancer in the future	0.74

The questionnaire form was checked by four academician nurses and three consultant surgeons for clarity and relevance of questions for the purpose of the study. Finally, 10 nurses and 10 teachers completed the questionnaire for pilot testing. They were asked to write down their views and suggestions regarding the questionnaire. Minor revisions resulted in the wording and style of the questions.

### Statistical analysis

The data were evaluated by descriptive statistics, factor analysis, chi-squared and *t-*tests. The results were expressed as mean ± standard deviation (SD), and differences between the groups were considered significant if the p value was less than 0.05. Missing data were deleted list-wise.

## Results

### Knowledge about BSE among nurses and teachers

The percentage of participants who had knowledge of BSE was higher in nurses than in teachers (81.5% versus 45.1%) (χ^2 ^= 39.039, p = 0.0001). The most common sources of information for nurses were written materials (42.6%), nursing school education (38.6%) and health professionals (37.6%). Television programs (56.1%), written material (38.3%) and health workers (35.6%) were the most frequent sources of information for teachers. However, 93.4% of the nurses and 98.2% of the teachers mentioned that they need more information about BSE.

A significantly greater percentage of nurses (69.3%) than teachers (46.7%) knew the correct timing of BSE for women with regular menstruation; χ^2 ^= 9.167, p = 0.002. However, nurses (20.4%) and teachers (24.6%) did not differ in their knowledge about the timing of BSE for women with irregular menstruations; χ^2 ^= 0.420, p = 0.517.

A significantly greater percentage of nurses (85.6%) than teachers (73.6%) said that they had knowledge about BSE; χ^2 ^= 3.908, p = 0.03. A significantly greater percentage of nurses (22.5%) than teachers (9.4%) gave correct answers to the question about the position for BSE; χ^2 ^= 3.893, p = 0.048. None of the participants from either group answered the question about the technique for BSE correctly.

### Attitudes of nurses and teachers to BSE

Table 4 shows the means and standard deviations of the BSE attitude factors in nurses and teachers. As in the table shows, nurses scored significantly higher than teachers on the "fear of breast cancer" factor only.

**Table 4:** The attitudes of the groups towards BSE

**Factors**	**Nurses**	**Teachers**	**t**	**p**
	**X ± SD**	**X ± SD**		
The importance of BSE in the early detection of breast cancer	4.36 ± 1.06	4.32 ± 0.91	.38	0.706
Fear of Breast cancer	3.96 ± 0.76	3.71 ± 0.93	2.49	0.01
Positive attitude for BSE	3.70 ± 0.63	3.83 ± 0.65	-1.60	0.109

### BSE practices of nurses and teachers

Significantly more nurses (73.5%) than teachers (42.5%) were self-confident about performing BSE; χ^2 ^= 27.045, p = 0.0001. More nurses (88.8%) than teachers (42.5%) said that they could easily recognize breast masses through BSE; χ^2 ^= 36.796, p = 0.0001. More nurses (48.8%) than teachers (10.4%) said that they have taught BSE to their patients or students χ^2 ^= 49.801, p = 0.000. Nurses said that they started performing BSE at the mean age of 22.57 ± 5.11 and teachers at the mean age of 32.87 ± 7.77; χ^2 ^= 26.652, p = 0.0001. Similar percentages of nurses (17.7%) and teachers (21.8%) said that they had performed BSE ten or more times during the past year; χ^2 ^= 0.411, p = 0.521. None of the participants were performing BSE properly (Table 5).

**Table 5:** Nurses' and teachers' BSE practice

**BSE practice**	**Nurses**		**Teachers**		χ^**2**^	**p**
	**n**	**%**^**a**^	**n**	**%**^**a**^		
Have you ever performed BSE?						
Yes	85	68.5	86	53.8	6.386	0.012
No	39	31.5	74	46.3		
When did you start BSE?						
≤ 19 age	31	45.6	4	6.2	26.652	0.000
> 19 age	37	54.4	61	93.8		
How often do you do BSE in one year?						
≥ 10 times	14	17.7	17	21.8	0.411	0.521
≤ 9 times	65	82.3	61	78.2		
When do you perform BSE?						
Two days after the cessation of menstruation	41	56.2	27	56.3	0.000	0.993
Any time	32	43.8	21	43.7		
What will be the position of body while performing BSE?						
True	16	21.3	9	13.6	1.426	0.232
False	59	78.7	57	86.4		
Which examination technique should be applied during BSE?						
True	-	-	-	-	-	-
False	70	100.0	55	100.0		

^a ^The percent of the column was mentioned.

### Why did nurses and teachers start performing BSE? Why did they not?

The fear of breast cancer was the leading motive for performing BSE among both nurses (42.3%) and teachers (52.3%) (Table 6). The most common reasons for not performing BSE in nurses were absence of complaints (82%), forgetting (56.4%), and anxiety about the possibility of recognizing a mass (28.2%). For the teachers, the most common reasons for not performing BSE were lack of knowledge (68.9%), absence of complaints (54%), forgetting (32.4%) and anxiety about finding a mass (37.8%).

**Table 6:** Why did nurses and teachers start performing BSE?

**Reasons for starting BSE**	**Nurses (n = 85)**		**Teachers (n = 86)**	
	**n**^a^	**%**	**n**^a^	**%**
Fear of breast cancer	36	42.3	45	52.3
Media	19	22.3	39	45.3
Doctor's advice	17	20.0	23	26.7
Breast pain	15	17.6	18	20.9
Advice of a health worker	9	10.5	4	4.6
Nipple discharge	5	5.8	9	10.4
The feeling of a mass	5	5.9	10	11.6
Breast cancer in the family	3	3.5	8	9.3
Encouraged by a friend	2	2.3	4	4.6
Others	25	29.4	4	4.6

^a ^More than one choice was indicated for the question.

## Discussion

It is widely accepted that nurses and teachers play important roles in establishing healthy behaviors. In particular, nurses play a primary role in increasing public awareness of breast cancer and BSE [30,40,41]. As expected, the nurses in our study were more aware than teachers about BSE (81.5% versus 45.1%).

Although some reports suggest that nurses are sufficiently skilled in performing and teaching BSE [12,42,43], others state that they might have inadequate information about breast cancer signs, screening methods and BSE [44]. Studies comparing the awareness of nurses and teachers about breast cancer have revealed that nurses are more capable [31]. Studies carried out in Turkey have revealed that 41.8% of Turkish women working in the health care sector know when to perform BSE and 67.I% know how to perform it [10,11].

Another important finding in our study was that knowledge about BSE among nurses and teachers was similar. The nurses gave more accurate answers only about the timing of BSE. Likewise, the position for BSE was correctly stated by nurses in an acceptable ratio. On the other hand, neither of the groups correctly answered the question about BSE technique.

The nurses often mentioned that their information sources were media and academic education [43,44]. In this study, the most important information sources for both our groups were written materials (books, magazines and booklets), academic education and health professionals. In addition, teachers support the idea that visual media tools such as television should be the leading sources of information.

### BSE Knowledge

Odusanya and Tayo [42] reported that Nigerian nurses were well-informed about breast cancer signs, diagnostic tests and BSE knowledge. Vurur et al. [43] showed that 86.3% of nurses inform patients correctly about the frequency of BSE, but fail to suggest the correct age to begin. Various studies have also stated that nurses and teachers lack knowledge about BSE [45,46]; in contrast, Franek et al. [44] reported that 63% of nurses knew almost everything about early breast cancer detection. Surprisingly, in this study, nurses knew less than expected about the position for BSE, although they had more positive answers about performing BSE.

Clarke and Savage (47) reported that BSE can be taught by a variety of professionals including nurses, physicians, trained peer educators, researches and graduate students without major differences. Heyman et al. [48] found that a program of instruction improved the abilities of nurses to teach BSE to their patients. Our findings strongly suggest that nurses and teachers should be encouraged with more theoretical background. In support of this result, both nurses and teachers were eager to learn more details about BSE.

### BSE Practice

It is reported that the ratio practicing BSE is low in teachers and health professionals [49]. Nurses present with a BSE practice ratio between 72.1% and 93%, increasing with age [12,50,51]. As expected, BSE practice among nurses was higher in this study (p = 0.012). In contrast to the findings of previous studies, nurses started BSE earlier in our group. An explanation may be that they began to receive information about BSE during nursing education.

In this study, more than half the nurses and teachers were performing BSE with appropriate timing. Consistent with our results, the timing of BSE was correct in 46% of the nurses who performed BSE monthly, as showed by Budden [50]. Here, the similarity in BSE practice between nurses and non-health care personnel should be considered. The teachers were more sensitive about BSE, possibly because of their age group, in which they are more likely to develop breast cancer.

The regularity of BSE among nurses in previous studies was between 18% and 67% [12,42,44,50]. Jarvandi et al. [49] found that 6% of Iranian teachers perform BSE regularly. The frequency of BSE in our groups was also low during the past year.

Heyman et al. [48] mentioned that 99% of nurses are self-confident about BSE, even though only 26% used the correct technique. In addition, Seif and Aziz [45] reported that the BSE technique of working women was positively altered by training. In our study, the nurses were convinced that they use the correct technique and can identify masses. On the other hand, neither of the groups performed BSE in the correct position. One surprising finding was that almost all participants performed BSE with the wrong technique. It is interesting to note that even the nurses made serious mistakes in BSE, although they were self-confident about their technique.

The incomplete knowledge of the nurses is important because 40% of them are the primary information sources for patients [40]. Budden [50] also reported that 77% of nurses relied on themselves to teach BSE but only 19% could teach BSE properly. Our study also revealed that nurses play their defined role in the community and teach BSE.

### BSE Attitudes

It has been reported that nurses and female health care workers have positive attitudes towards early detection of breast cancer [45,51]. Jarvandi et al. [49] stated that most teachers feel themselves at risk of breast cancer. Because of this, they perform BSE routinely. In the present study, both groups had positive attitudes to BSE and were convinced of its value for early diagnosis of breast cancer. Our findings showed that both groups cared about BSE. Another finding supporting this was the low ratio of average factor points in both nurses and teachers.

Interestingly, the mean score on the "Fear of breast cancer" factor was higher among nurses than teachers. This may be explained by the nurses' working environment, in which they take care of patients with breast cancer.

## Conclusion

Knowledge about BSE was found to be acceptable in both groups. However, BSE technique (i.e. correct posture during examination) was poor. Both groups presented with positive attitudes towards BSE. Nurses begin BSE earlier and rely on their skills to perform it. However, none of the groups performed BSE regularly and they had problems with the correct position and palpation technique.

Since nurses' and teachers' beliefs and behaviors may have an impact on young females, it is essential to plan training courses for this group of women. Therefore, continuing education and in-service education for nurses and teachers should be planned to improve their knowledge and experience of BSE, because of their professional roles in community awareness about breast cancer and screening methods.

## Competing interests

The author(s) declare that they have no competing interests.

## Authors' contributions

The research was planned end conducted by FD. FD and PT performed the statistical analyses and contributed to discussing the results and drafting the manuscript. NAB and SM contributed to data analyses and drafting of the manuscript.GT and SÖ collected the data.

## Pre-publication history

The pre-publication history for this paper can be accessed here:



## References

[B1] Jemal A, Siegel R, Ward E, Murray T, Xu J, Smigal C, Thun MJ (2005). Cancer statistics 2006. CA: A Cancer J Clin.

[B2] Anderson BO, Shyyan R, Eniu A, Smith RA, Yip CH, Bese NS, Chow LW, Masood S, Ramsey SD, Carlson RW (2006). Breast cancer in limited-resource countries: An overview of the Breast Health Global Initiative 2005 Guidelines. Breast J.

[B3] Groot MT, Baltussen R, Uyl-de Groot CA, Anderson BO, Hortobágyi GN (2006). Costs and health effects of breast cancer interventions in epidemiologically different regions of Africa, North America, and Asia. Breast J.

[B4] American Cancer Society (2002). *Cancer Facts and Figures 2002*.

[B5] Dorey E (2004). Running from breast cancer. Cancer Nurs Pract.

[B6] T.R. Ministry of Health (2000). Cancer control programme and cancer statistical in Turkey (1995–1999).

[B7] Fidaner C, Eser SY, Parkin DM (2001). Incidence in İzmir in 1993–1994: first results from Izmir Cancer Registry. Eur J Cancer.

[B8] Parlar S, Bozkurt Aİ, Ovayolu N (2004). Ana Çocuk Sağlığı ve Aile Planlama merkezine başvuran kadınlarda kendi kendine meme muayenesi ile ilgili bilgi, tutum ve davranışların değerlendirilmesi. Sağlık ve Toplum.

[B9] Fındık ÜY, Turan N (2004). Kadınların meme kanserinin erken tanısına yönelilk davranışlarının belirlenmesi. Hemşirelik Forumu, Kasım-Aralık.

[B10] Kırdök E, Budakoğlu Iİ, Maral I (2004). Hekimdışı sağlık personelinin meme kanseri hakkındaki bilgi ve davranışları. Sağlık ve Toplum.

[B11] Öncel S, Özdemir Akcan A (2004). Kadınların meme kanseri ve kendi kendine meme muayenesi konusundaki bilgilerinin incelenmesi. MN-Klinik Bilimler & Doktor.

[B12] Chong PN, Krishnan M, Hong CY, Swash TS (2002). Knowledge and practice of breast cancer screening amongst public health nurses in Singapore. Singapore Med J.

[B13] American Cancer Society (2005). *Breast Cancer Facts & Figures 2005–2006*. American Cancer Society Inc Atlanta.

[B14] Anderson BO, Braun S, Carlson RW, Gralow JR, Lagios MD, Lehman C, Schwartsmann G, Vargas HI (2003). Overview of breast health care guidelines for countries with limited resources. Breast J.

[B15] Duffy SW, Tabar L, Vitak B, Warwick J (2006). Tumor size and breast cancer detection: What might be the effect of a less sensitive screening tool than mammography?. Breast J.

[B16] Canadian Cancer Society/National Cancer Institute of Canada (2006). Canadian Cancer Statistics Toronto.

[B17] Zincir H (1999). Malatya il merkezinde 40 yaş üzeri kadınların meme kanseri ve korunma konusunda bilgi tutum ve davranışları. MSc Thesis.

[B18] T.R. Ministry of Health (1999). The report for the control program of breast cancer: Follow-up report for breast self-examination 2004. http://www.saglik.gov.tr/extras/birimler/ksdb/2.doc.

[B19] Çeber E, Soyer MT, Ciceklioglu M, Cimat S (2006). Breast cancer risk assessment and risk perception on nurses and midwives in Bornova Health District in Turkey. Cancer Nurs.

[B20] Loescher L (2004). Nursing roles in cancer prevention position statements. Semin Oncol Nurs.

[B21] Smith RA, Cokkinides V, Eyre HJ (2005). American Cancer Society Guidelines for the Early Detection of Cancer, 2005. CA: A Cancer J Clin.

[B22] Abdel-Fattah M, Zaki A, Bassili A, el-Shazly M, Tognoni G (2000). Breast self-examination practice and its impact on breast cancer diagnosis in Alexandria, Egypt. East Mediterr Health J.

[B23] Kern KA (1994). Medicolegal analysis of the delayed diagnosis of cancer in 338 cases in the United States. Arch Surg.

[B24] Law M (2004). Screening without evidence of efficacy. Br Med J.

[B25] Kösters JP, Gøtzsche PC (2003). Regular self-examination or clinical examination for early detection of breast cancer. Cochrane Database Syst Rev.

[B26] Smith RA, Cokkinides V, Eyre HJ (2006). American Cancer Society Guidelines for the Early Detection of Cancer, 2006. CA: A Cancer J Clin.

[B27] Canadian Cancer Society/National Cancer Institute of Canada (2005). Canadian Cancer Statistics 2005. Toronto.

[B28] Goyal V (2001). Self breast examination (letter). Br Med J.

[B29] Howard F, Scott-Findlay S (2006). Breast self-examination when research contradicts accepted practice. AWHONN Lifelines.

[B30] Bailey K (2000). The nurse's role in promoting breast awareness. Nurs Stand.

[B31] Madanat H, Merrill RM (2002). Breast cancer risk-factor and screening awareness among women nurses and teachers in Amman, Jordan. Cancer Nurs.

[B32] Leslie NS (1995). Role of the nurse practitioner in breast and cervical cancer prevention. Cancer Nurs.

[B33] Spratt J, Shucksmith J (2006). Tobacco education in the primary school: Paradoxes for the teacher. Health Educ J.

[B34] Sapien RE, Fullerton-Gleason L, Allen N (2004). Teaching school teachers to recognize respiratory distress in asthmatic children. J Asthma.

[B35] Ford T, Nikapota A (2000). Teachers'attitudes towards child mental health services. Psychiatric Bull.

[B36] Mathews C, Boon H, Flisher AJ, Schaalma HP (2006). Factors associated with teachers' implementation of HIV/AIDS education in secondary schools in Cape Town, South Africa. AIDS Care.

[B37] Budden L (1999). Student nurses' breast self-examination health beliefs, attitudes, knowledge, and performance during the first year of a pre-registration degree program. Cancer Nurs.

[B38] T.R Ministry of Health (2005). National Guideline for Family Planning and Reproductive Health.

[B39] Smith RA, Saslow D, Sawyer KA, Burke W, Costanza ME, Evans WP, Foster RS, Hendrick E, Eyre HJ, Sener S (2003). American Cancer Society guidelines for breast cancer screening: update 2003. CA Cancer J Clin.

[B40] Clarke DE, Sandler LS (1989). Factors involved in nurses' teaching breast self-examination. Cancer Nurs.

[B41] Peragallo NP, Fox PG, Alba ML (2000). Acculturation and breast self-examination among immigrant Latina women in the USA. Int Nurs Review.

[B42] Odusanya OO, Tayo OO (2001). Breast cancer knowledge, attitudes and practice among nurses in Lagos, Nigeria. Acta Oncologia.

[B43] Vurur S, Kaya F, Ünüvar R, Sezgin H The evaluation of knowledge and behaviours of nurses and midwives on breast self-examination. Presented at 4th international reproductive health and family planning congress: 20–23 April 2005.

[B44] Franek GA, Nowak-Kapusta ZE, Cabaj M (2004). Breast cancer prophylaxis among nurses. Wiad Lek.

[B45] Seif NY, Aziz MA (2000). Effect of breast self-examination training program on knowledge, attitude and practice of a group working women. J Egypt Natl Canc Inst.

[B46] Odusanya OO (2001). Breast cancer: Knowledge, attitudes, and practices of female schoolteachers in Lagos, Nigeria. Breast J.

[B47] Clarke VA, Savage SA (1999). Breast self-examination training: A brief review. Cancer Nurs.

[B48] Heyman E, Tyner R, Phipps C, Cave L, Owen DC (1991). Is the hospital setting the place for teaching breast self-examination?. Cancer Nurs.

[B49] Jarvandi S, Montazeri A, Harirchi I, Kazemnejad A (2002). Beliefs and behaviours of Iranian teachers toward early detection of breast cancer and breast self-examination. Public Health.

[B50] Budden L (1998). Registered nurses' breast self-examination practice and teaching to female clients. J Commun Health Nurs.

[B51] Haji-Mahmoodi M, Montazeri A, Jarvandi S, Ebrahimi M, Haghighat S, Harirchi I (2002). Breast self-examination: Knowledge, attitudes, and practices among female health care workers in Tehran, Iran. Breast J.

